# The Use of Carbolic Acid by Subcutaneous Injection, in the Treatment of Erysipelas

**Published:** 1878-03

**Authors:** James S. Whitmire

**Affiliations:** Metamora, Ills.


### THE USE OF CARBOLIC ACID BY SUBCUTANEOUS INJECTION, IN THE TREATMENT OF ERYSIPELAS.

By James S. Whitmire, M. D., Metamora, Ills.

Erysipelas may occur from a variety of causes, such as traumatism, specific or local influence, and also from general or specific fevers, the origin of which may be found in the sporules of microscopic fungi, or germs, that find their way into the circulation by means of respiration, food, drink, wounds, etc. —thus engendering a portion at least, of our so-called zymotic diseases. In either case, the cause of the local inflammation, though there may be a difference in the grade, is, evidently, derived from some septic or specific source. Without any intention of writing a lengthy dissertation on either the etiology or pathology of erysipelas, I have made the foregoing concise statements regarding the disease, because they seem to be in accord with generally accepted theory, and because what I shall further say respecting treatment, will thus seem more clear and rational to the reader. It is now a pretty generally conceded fact, that in the treatment of erysipelas, medicines of a sustaining character are required, to successfully combat this disease, as well as those that antagonize the zymoma. Hence. the rationale of the use of the tinct. ferri chloridi in erysipelas: because, by its absorption into the circulation, it enables the red corpuscles of the blood to carry a greater amount of oxygen to the starving tissues; and the presence of the iron in the blood gives to this fluid a richness, and we might say a tonicity, that probably intercepts the fermentive process that has been previously occasioned by septic influences. Besides this, it is more than probable that the small excess of hydrochloric acid contained in this tincture, may, and probably does, conduce to the same end. Again, the potassic chlorate is also considered a valuable remedy in the disease, and this, more than likely, on account of the large proportion—39 per cent.—of oxygen carried with it into the circulation for the revivifying of tissues and the facilitating healthy molecular change. Quinine is also given, not alone on the theory that it is destructive to the lower organisms, but that it is both a febrifuge and a tonic, and therefore, a supporter of the vital functions.

My object in this communication is simply to give succinctly my clinical experience, and also to make some suggestions regarding the physiological action of carbolic acid, when used as a subcutaneous injection in erysipelas. Some time before using carbolic acid in this manner, my attention had been called to the probability of its favorable action by subcutaneous injection in erysipelatous inflammation, by a simple paragraph in one of the medical journals. This paragraph made an impression on my mind, as I recognized the parasiticidal effect of the drug and its acknowledged anti-zymotic action. I was thus prepared at the first favorable opportunity to investigate its adjuvant action in connection with other and established remedies. I reasoned in this way: besides the antiseptic properties that the acid is known to possess, in its pure state, it is an escharotic; so also are iodine, bromine, and the mineral acids; and yet, in a dilute condition, they manifest very different and valuable properties, and become not only harmless in their action upon the economy, but tonic, alterative, rubefacient, deturgent, or absorbent, according to the condition for which either of them may be used. I argued that owing to its caustic action in a pure state, it might act as a simple local stimulant when subcutaneously injected in a dilute form. Why should it not stimulate the capillary vessels of the part, and enable them to relieve themselves of their overloaded contents? Might it not also stimulate the absorbent or lymphatic vessels, and enable them to take up effused serum escaped from engorged capillaries into the cellular tissue? Thus reasoning, I was prepared to test the acid, and in a short time after I had come to this conclusion, an opportunity offered in the case of an old German lady, aged 70 years, as I have since learned. I was called to see her July 6th, 1876. I reported this case, verbally, to the Illinois State Medical Society, at its meeting in Chicago, May, 1877. I found one eye sealed, on account of swelling. The inflammation extended over the scalp to the ear, and over the face to the mouth. The swelling was purple from engorgement of capillaries, and vesication had taken place at two or three points. I thought it would slough, and I came to this conclusion more on account of her advanced age and the feeble condition of her general health, than from any other reason. It was certainly a case that would result in gangrene under any of the ordinary methods of treatment.

Here was an old lady who, from the infirmities of age, could not be cheated, under any circumstances, out of many years. But I had faith in my theoretic reasoning and the knowledge that the acid, by its absorption into the general circulation, would come unchanged into direct contact with microscopic germs or other septic agents which it would destroy. Thus the blood would be depurated and the nervous system relieved of a corresponding cause of depression. Since verbally reporting the above case at the Illinois Medical Society, I have formulated my prescription, so that others who have no experience with the subcutaneous use of this acid may employ it without the remotest risk of doing mischief. The subjoined formula is of about the same strength as that employed in the case referred to above :—

Carbolic acid (crystals), 1 ounce avoirdupois, pure glycerine, fld.℥j., mix and warm in a water bath till the acid is dissolved. To prepare this fluid for the purpose of injection I use the following proportions : glycerine, ℥ss., water ℥ss., of the mixture described above, drops xx. There are therefore, about ten drops of the pure acid to each fluid ounce of the mixture, or injection fluid, and therefore one and a quarter ($1\frac{1}{4}$) drops to each drachm of the fluid. By count, this mixture contains about sixty (60) drops to the drachm, so that it is an easy matter to know exactly what amount of the pure acid is used at any given time. In the case of the woman alluded to, I used two drachms of the latter mixture the first time, which would be equivalent to two and one half drops of the pure acid. I introduced the point of the syringe at a dozen or more points, completely encircling the discolored skin, and at a half dozen or more points over the diseased surface; after which, to keep the surface moist, I used a solution of iodine in castor oil, the formula for which I here give: iodine, gr. xv., iod. pot., gr. x., alcohol, ʒjj., rub thoroughly in a Wedgewood mortar, add gradually, castor oil, ℥iv., till the iodine is all dissolved. In a few minutes is formed a fine red solution of iodine. I prefer this solution lest a stronger one produce an irritating or vesicant effect upon the skin, which would rather favor the spread of the local affection than otherwise. I ordered the following prescription to be taken internally: ℞. tinct. ferri. chlorid., f.℥ss., pot. chlor., ʒij., ammoniæ hydrochlo., ʒjjj., syrup. simp., ad f.ʒiv.; of this mixture one tablespoonful is to be taken in water every four hours. I also ordered; ℞. opii. pulv., gr., iij., quin. sulph., ʒss., pot. chlor., ʒj.; M. Div. in pulv. ix—Sig.: One to be taken between each dose of the mixture. Under this treatment, in twenty-four hours, the swelling had considerably abated so that she could freely open her eyes.

July 7.—Temp., 100° F.; pulse, 98; respirations, 20 per m. I repeated the injections, and continued treatment.

July 8.—Swelling still abating; she opens her eyes much better; the disease not disposed to spread. Temp., 98° F.; pulse, 90; respiration natural. I gave another injection, and ordered medicine at longer intervals. On the 9th I used the last subcutaneous injection; Temp. 98° F.; pulse, 84. I continued the medicines at intervals of five hours, with the powders lessened in quantity, and left enough to last her several days, but did not visit her again. On the fourth day the erysipelatous appearance hadvery nearly all left her face and scalp, so that she was looking quite natural, excepting the three places where the cuticle had been loosened from the disease previous to my first visit. During the four days I attended this lady, she had very little fever, and the only grave symptom manifested was a mild, wandering delirium during the first three nights, after which she was not troubled in that way. This nocturnal delirium had been present previous to my having seen her, else I might have attributed it either to the opium or carbolic acid.

I have been thus explicit in my report of this case that others may know exactly how to go to work in similar cases, and, also, that in my report of the two following cases I would not have to be so particular. I have never used the carbolic acid injection when other means were not used at the same time in conjunction with it. I have been afraid to take the chances; but that it is a very valuable adjuvant in the treatment of erysipelas, I no longer doubt. My note book at this date, January 3, 1878, now shows thirty cases treated subcutaneously in the same manner, including those formerly reported to the Illinois State Medical Society.

Case II. Mrs. M., German, a farmer's wife, aged 50. I was called to see her November 13th, 1877. I was told by the messenger who came for me, that "her face was as red as a rose, and her head as big as a bushel." I at once suspected the trouble, and therefore went prepared with the requisite materials. I found the woman truly in a critical condition. The erysipelas had commenced on the morning of the 10th, and rapidly progressed till her whole face and scalp had become involved. In her case I used nearly three drachms of the dilute acid, as a subcutaneous injection, and gave her the same prescription for internal use, as in the former case, with oleaginous solution of iodine as an embrocation to the skin. On the 14th, I was unable to visit my patient on account of indisposition; but my son, Dr. J. W. Whitmire, went to see her. He reported the pulse, 90; temperature, 100; not so much thirst, and the swelling somewhat reduced. He used two drachms of the dilute acid, and continued the same prescription, only ordering that if she slept at night, she should not be awakened. Nov. 15th, my son reported the case progressing favorably; used one and a half drachms of the dilute acid; continued former treatment. Two more visits were made, one on the 17th, the other on the 19th; at each visit the subcutaneous and other treatment was continued, only lengthening the interval of taking the iron mixture to five hours. At the last visit the patient was supplied with medicine to last for three or four days, and discharged convalescent. It must be remembered that the bottle containing the dilute acid for injection, was always set in a tin of hot water before use, and brought to a temperature of 98° F.

Case III. My last case of erysipelas was Mr. G. E.; sanguine temperament; American; carpenter; age, 56. He came to my office December 15th, 1877. He said his forehead and scalp as far back as the crown and back of the left ear, had been burning hot, and kept him uneasy during the whole previous night. There was some tumefaction and soreness, and the erysipelatous blush was very perceptible, so that the diagnosis was easily made. The disease was fairly in the process of development. I introduced the subcutaneous injection round the seat of disease and over the discolored skin, from the eyebrow to the region behind the ear. I used the oleaginous solution of iodine over the affected part, and gave: ℞ tinct. ferri chlorid. ʒiv., ammon. hydro. chlor., pot. chlor. āā ʒjj., quin. sulph. ʒss., syrup. simp. ad. f℥iv. Of this mixture he was ordered one tablespoonful diluted with water, during the day time, and also at night if he awoke. On the 16th, the swelling was so much reduced, that I did not resort to the injection again. I kept him on the use of the mixture, however, with the iodized oil for four or five days, when the discoloration and swelling had entirely disappeared, and my patient was well. This case is reported more particularly, because it is one of absolutely aborted erysipelas, and is a correct representative of nearly all of the thirty (30) cases that I have treated, in the interim, between it and the first case in which I used carbolic acid injections. Many of the cases which I have seen early, have been brought, I might say, to an almost sudden termination by the immediate application of the carbolic acid treatment; and in no single instance have I seen an untoward symptom arise from the tonic effects of the acid, not even when in one case I must have used, at least, five drops of the pure acid in a dilute state. Many of my patients complained, after its use, of a certain amount of numbness, or want of feeling in the parts subjected to its use. This I considered an anæsthetic effect due to the acid. In no case in which I have used this injection, has there been a sore or abscess formed, so that I may say that I have found carbolic acid, not only a safe and very valuable remedy, but almost a specific in the treatment of erysipelas. It seems to me that the thirty cases in which I have used this remedy so successfully, should be quite sufficient to recommend it to the favorable consideration of the profession.

